# Endoscopic Nd:YAG Laser Treatment in the Perioperative Management of Tracheobronchoplasty

**DOI:** 10.1155/DTE.3.53

**Published:** 1996

**Authors:** Takehiko Fujisawa, Yukio Saitoh, Masayuki Baba, Mitsutoshi Shiba, Yasuo Sekine, Tsunehiro Takeda, Shigetoshi Yoshida, Yutaka Yamaguchi

**Affiliations:** Department of Surgery Institute of Pulmonary Cancer Research Chiba University School of Medicine 1-8-1 Inohana, Chuo-ku Chiba 260 Japan

## Abstract

The objective of this study was to determine the role of endoscopic Nd:YAG laser treatment in the preoperative or postoperative management of tracheobronchoplasty. Eighteen patients with severe stenotic lesions of the trachea or bronchus underwent Nd:YAG laser treatment. Nd:YAG laser treatment was performed in the preoperative period in 14 patients and in the postoperative period in 4 patients. The indications for Nd:YAG laser treatment included emergency airway dilatation, confirmation of the distal margin of tumor, and safe tracheal intubation in patients with severe tracheal stenosis. The indications for Nd:YAG laser treatment in patients with severe stenosis of the mainstem bronchus were confirmation of the distal margin of tumor and recovery of lung ventilation during the preoperative period and reopening of the bronchial lumen to prevent obstructive pneumonia in the postoperative period. Among patients treated with Nd:YAG laser preoperatively, the indications were completely achieved in all 14 patients, except for 1 patient with adenoid cystic carcinoma who underwent treatment of the right mainstem bronchus. Among patients treated with Nd:YAG laser postoperatively the indications also were achieved in all 4 patients with severe granulomatous stenosis of the bronchial end-to-end anastomosis following sleeve lobectomy. In conclusion, endoscopic Nd:YAG laser treatment played an important role in the perioperative management of patients undergoing tracheobronchoplasty.


*Diagnostic and Therapeutic Endoscopy*, Vol. 3, pp. 53–59
Reprints available directly from the publisher
Photocopying permitted by license only

© 1996 OPA (Overseas Publishers Association)
Amsterdam B.V. Published in The Netherlands
by Harwood Academic Publishers GmbH
Printed in Singapore

### Endoscopic Nd:YAG Laser Treatment in the Perioperative Management of Tracheobronchoplasty

TAKEHIKO FUJISAWA*, YUKIO SAITOH, MASAYUKI BABA, MITSUTOSHI SHIBA, YASUO SEKINE, TSUNEHIRO TAKEDA, SHIGETOSHI YOSHIDA and YUTAKA YAMAGUCHI

*Department of Surgery, Institute of Pulmonary Cancer Research, Chiba University School of Medicine, 1-8-1 Inohana, Chuo-ku, Chiba 260, Japan*

*(Received 29 January 1996; In final form 25 March 1996)*

**The objective of this study was to determine the role of endoscopic Nd:YAG laser treatment in the preoperative or postoperative management of tracheobronchoplasty. Eighteen patients with severe stenotic lesions of the trachea or bronchus underwent Nd:YAG laser treatment. Nd:YAG laser treatment was performed in the preoperative period in 14 patients and in the postoperative period in 4 patients. The indications for Nd:YAG laser treatment included emergency airway dilatation, confirmation of the distal margin of tumor, and safe tracheal intubation in patients with severe tracheal stenosis. The indications for Nd:YAG laser treatment in patients with severe stenosis of the mainstem bronchus were confirmation of the distal margin of tumor and recovery of lung ventilation during the preoperative period and reopening of the bronchial lumen to prevent obstructive pneumonia in the postoperative period. Among patients treated with Nd:YAG laser preoperatively, the indications were completely achieved in all 14 patients, except for 1 patient with adenoid cystic carcinoma who underwent treatment of the right mainstem bronchus. Among patients treated with Nd:YAG laser postoperatively the indications also were achieved in all 4 patients with severe granulomatous stenosis of the bronchial end-to-end anastomosis following sleeve lobectomy. In conclusion, endoscopic Nd:YAG laser treatment played an important role in the perioperative management of patients undergoing tracheobronchoplasty.**

*Keywords:* Bronchofiberscopy, Nd:YAG laser, tracheal stenosis, lung cancer, bronchoplasty

### INTRODUCTION

Tracheobronchoplasty has become an established operative method for the preservation of lung function in patients with benign or malignant diseases of the trachea or bronchus [1,2]. Severe tracheal stenosis, however, may require urgent airway reopening as a lifesaving measure. Stenosis of the anastomotic site of the tracheobronchoplasty also may occur. The optimal management modality for these perioperative problems remains to be determined [3].

*Corresponding author. Tel.: 043-222-7171, ext. 5462. Fax: 043-226-2172.

In this study, we evaluated the role of endoscopic Nd:YAG laser treatment in the perioperative management of tracheobronchoplasty in patients with benign and malignant tracheobronchial stenosis.

### MATERIALS AND METHODS

### Patients

Among 50 patients undergoing tracheal or bronchial plasty, 18 patients were treated with endoscopic Nd:YAG laser treatment preoperatively or postoperatively and were included in this study (Table I). The operation performed for tracheobronchoplasty consisted of tracheal sleeve resection with end-to-end anastomoses in 8 patients, sleeve lobectomies in 8 patients, sleeve lobectomy and carinal reconstruction in 1 patient, and bronchial sleeve resection in 1 patient.

### Method of Nd:YAG Laser Irradiation

Endoscopic Nd:YAG laser treatment was performed according to the method previously described [4]. Nd:YAG laser (Medilas YAG, MBB Co., Germany) treatment was performed by a noncontact method at approximately 30 to 50 W for 1 sec intermittently through a quartz fiber covered with a Teflon tube 2.0 mm in diameter inserted via a biopsy channel of a bronchofiberscope. All patients were treated under local anesthesia with 10 ml of 4% lidocaine hydrochloride after administration of 0.5 mg of atropine sulfate and 15 to 30 mg of pentazosin. When Nd:YAG laser treatment was performed for severe tracheal stenosis, tumor vaporization was performed at the same time from the surface of the airway. During the first Nd:YAG laser treatment, an attempt to reopen the tracheal lumen to greater than 6.0 mm in diameter so that a bronchoscope of 6.0 mm in outer diameter could be placed to relieve wheezing and dyspnea. The tracheal lumen was reopened to 8 to 10 mm in diameter during the following two to five sessions of Nd:YAG laser treatment.

TABLE I Patient Characteristics

| Characteristic | No. of Patients |
|---|---|
| Age (mean): 11–75 yr (52) | |
| Sex | |
| men | 13 |
| women | 5 |
| Histology | |
| ACC | 5 |
| SCC | 4 |
| Carcinoid | 1 |
| Adenocarcinoma | 1 |
| Granulation | 7 |
| Timing of Nd:YAG | |
| Preoperative | 14 |
| Postoperative | 4 |
| Site of Nd:YAG | |
| Trachea | 8 |
| Bronchi | 10 |

ACC, adenoid cystic carcinoma; SCC, squamous cell carcinoma

### Indications for and Effects of Nd:YAG Laser Treatment

In patients with severe tracheocarinal stenosis, indications for preoperative Nd:YAG laser treatment included lifesaving airway reopening, confirmation of the distal margin of tumor, and safe endotracheal intubation for general anesthesia. In patients with bronchial stenosis, indications for preoperative Nd:YAG laser treatment consisted of airway reopening for the confirmation of the distal margin of tumor and recovery of ventilation. In patients treated with the Nd:YAG laser postoperatively, airway reopening was the indication. Efficacy was defined as "effective" when the indications were achieved, with reopening of lumen diameter 6 mm or more and improved lung function parameters, and "ineffective" when the indications were not achieved, without reopening of lumen diameter 6 mm or more and without improved lung function parameters.

### RESULTS

Of the 8 patients treated with the Nd:YAG laser preoperatively for tracheocarinal stenosis (Table II), the chief complaints were severe dyspnea and wheezing.

TABLE II Improvement of Lung Ventilation by Endoscopic Nd:YAG Laser Treatment in Patients with Tracheoplasty or Tracheocarinal Plasty

| Patients | Age (yr)/Sex | Histology | No. Sessions | Total J | FVC (liters) | | FEV1/FVC% | | FEV1 (liters) | | PEFR (liters/sec) | | Effect | Survival |
|---|---|---|---|---|---|---|---|---|---|---|---|---|---|---|
| | | | | | Before | After | Before | After | Before | After | Before | After | | |
| I.T. | 56/F | ACC | 2 | 20,757 | 2.86 | 2.68 | 25 | 80.5 | 0.73 | 2.12 | 0.83 | 4.29 | Effective | 120 mo alive |
| M.T. | 45/F | ACC | 2 | 12,775 | 2.16 | 2.87 | 11.6 | 45.6 | 0.31 | 1.23 | 0.48 | 1.25 | Effective | 66 mo alive |
| H.W. | 64/M | SCC | 2 | 11,095 | 1.9 | 2.97 | 21 | 72 | 0.4 | 2.38 | 0.96 | 5.04 | Effective | 60 mo alive |
| T.U. | 75/F | ACC | 2 | 8,043 | 1.88 | 2.21 | 66.5 | 79.6 | 1.25 | 1.76 | 1.21 | 4.34 | Effective | 54 mo alive |
| T.S. | 65/F | ACC | 2 | 12,334 | 1.42 | 2.26 | 90.1 | 99.6 | 1.29 | 2.25 | 1.92 | 3.87 | Effective | 48 mo alive |
| O.K. | 13/M | Granulation | 5 | 18,707 | 2.01 | 2.53 | 81 | 74.3 | 1.62 | 1.88 | 1.67 | 2.1 | Effective | 102 mo alive |
| S.K. | 11/M | Granulation | 3 | 4,731 | 3.1 | 3.48 | 19.3 | 40.2 | 0.6 | 1.4 | 0.6 | 1.46 | Effective | 72 mo alive |
| O.H. | 34/M | Granulation | 6 | 19,981 | 3.21 | 3.36 | 68.2 | 82.7 | 2.19 | 2.78 | 2.08 | 4.62 | Effective | 51 mo alive |
| Mean | | | 3 | 13,553 | 2.32 | 2.8* | 47.8 | 71.8* | 1.05 | 1.98** | 1.22 | 3.37** | | |

*$P < 0.1$,

**$P < 0.01$ compared with the value before Nd:YAG laser therapy, FVC, forced vital capacity; FEV, forced expiratory volume; PEFR, peak expiratory flow rate.

Significant improvements of forced expiratory volume 1.0 and peak expiratory flow rate and possible improvement of forced vital capacity and forced expiratory volume 1.0% were demonstrated. The indications for endoscopic Nd:YAG laser treatment were completely achieved in all 8 patients. All patients were alive and were able to resume good daily life activity.

Among 6 patients with bronchial stenosis (Table III), the indications for endoscopic Nd:YAG laser treatment were achieved in 5 patients. However, in a patient with adenoid cystic carcinoma, continuous bleeding from the irradiated sites forced the cessation of Nd:YAG laser treatment, and bleeding stopped spontaneously approximately 5 minutes later. The patient underwent right sleeve pneumonectomy and patient died from dehiscence of the anastomosis between the trachea and left mainstem bronchus 1 month postoperatively.

In patients treated with the Nd:YAG laser postoperatively (Table IV), significant improvement of airway reopening was demonstrated in all 4 patients. Two patients died at 12 and 36 months after Nd:YAG laser treatment, and the other 2 patients were alive without restenosis of the anastomotic site at 5 and 7 years after Nd:YAG laser treatment. No complications occurred except for a patient with continuous bleeding in this series of the study.

### Case Report

T.S., a 65-year-old woman was seen with chief complaints of wheezing, dyspnea, and bloody sputum. In April 1991, her symptoms gradually worsened, and she was admitted to our department in May. Bronchofiberscopic findings revealed a polypoid type tumor occupying approximately 90% of the tracheal lumen at the first cartilaginous ring below the vocal cord (Fig. 1A). Two sessions of endoscopic Nd:YAG laser treatment (first: 6010 J, second: 6324 J) were performed as an emergency treatment. The tracheal lumen was reopened to 10 mm (Fig. 1B), and her symptoms completely disappeared. The longitudinal distance of invasion along the trachea was 3.5 cm, and the distal margin of tumor was located at the 13th cartilaginous ring from the carina. Biopsy specimens revealed adenoid cystic carcinoma. Induction for general anesthesia was performed with routine muscle relaxants, and a spiral tube (12 mm in outer diameter) was intubated intratracheally without difficulty. Four centimeters of trachea including six cartilaginous rings were resected, and an end-to-end anastomosis was performed with 3-0 absorbable synthetic sutures. Pathologic examination demonstrated adenoid cystic carcinoma with residual cancer cells at the resected superior margins. The postoperative course was uneventful, and a total of 60 Gy of radiotherapy was

TABLE III Endoscopic Nd:YAG Laser Surgery Before Sleeve Lobectomy or Sleeve Pneumonectomy

| Patient | Age (yr)/Sex | Histology | No. Sessions | Total J | Effectiveness | Survival |
|---|---|---|---|---|---|---|
| K.M. | 42/M | Adenocarcinoma | 2 | 7,904 | Effective | 8 mo dead |
| F.M. | 67/M | SCC | 1 | 4,850 | Effective | 2 mo dead |
| T.A. | 65/F | ACC | 1 | 715 | Ineffective | 1 mo dead |
| T.M. | 41/M | SCC | 1 | 1,888 | Effective | 7 yr alive |
| S.M. | 69/M | SCC | 2 | 10,547 | Effective | 3 mo alive |
| I.T. | 36/M | Carcinoid | 4 | 25,206 | Effective | 30 mo alive |

TABLE IV Endoscopic Nd:YAG Laser Surgery After Sleeve Lobectomy

| Patient | Age (yr)/Sex | Disease | No. Sessions | Total J | Effectiveness | Survival |
|---|---|---|---|---|---|---|
| T.K. | 56/M | Granulation | 3 | 4,347 | Effective | 36 mo dead |
| T.S. | 65/M | Granulation | 3 | 12,787 | Effective | 60 mo alive |
| M.K. | 53/M | Granulation | 2 | 2,624 | Effective | 48 mo alive |
| S.A. | 72/M | Granulation | 1 | 338(CT) | Effective | 12 mo dead |

CT, contact tip.

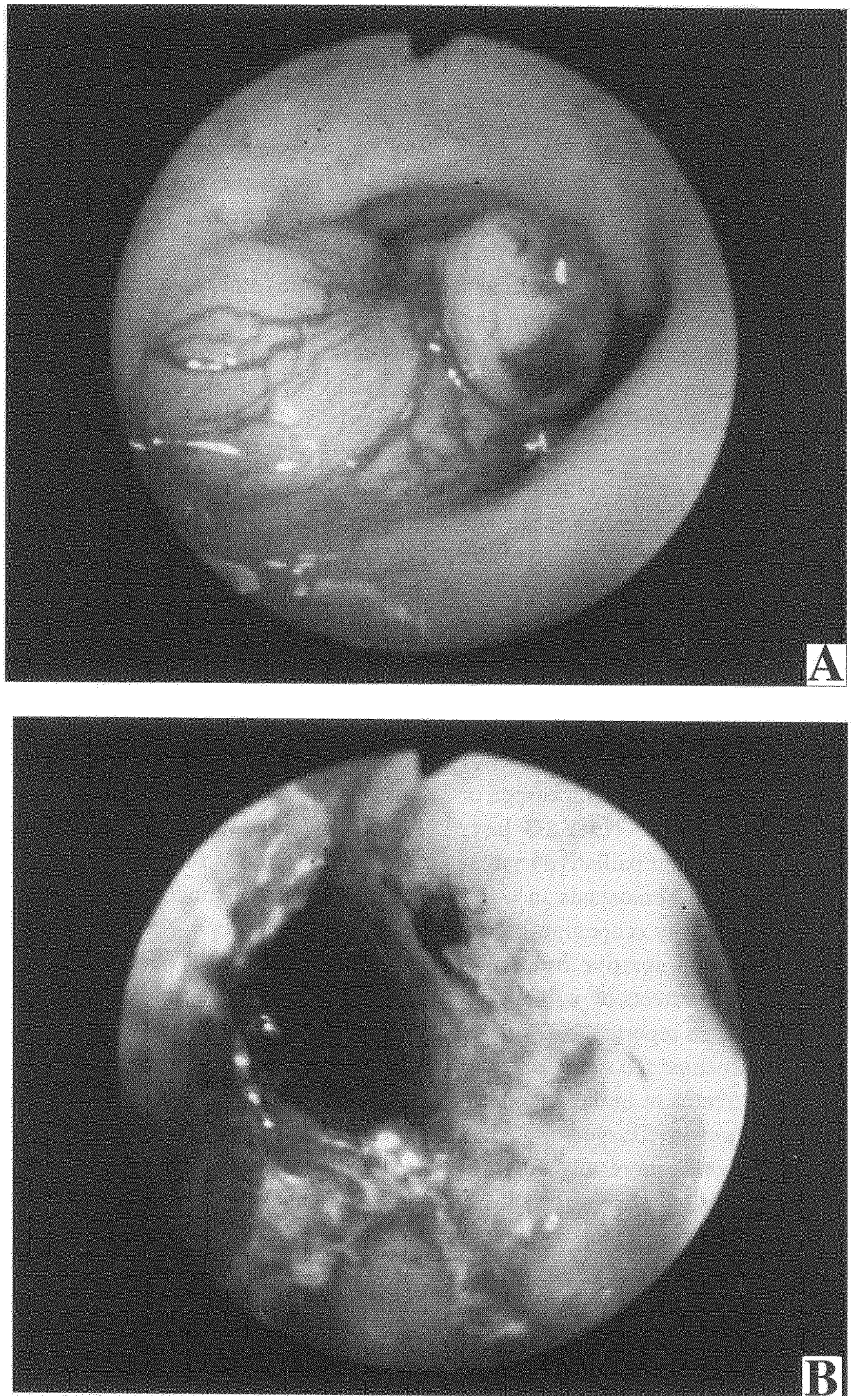

FIGURE 1 A. Bronchofiberscopic findings revealed a protruding tumor with superficial blood vessel dilatation obstructing approximately 90% of the tracheal lumen. B. After two sessions of Nd:YAG laser treatment, the tracheal lumen was reopened to approximately 10 mm in diameter. Symptoms completely disappeared, and the distal margin of tumor at the 13th cartilaginous ring from the carina was confirmed by bronchoscopy.

added postoperatively. The patient is in good physical condition without any clinical sign of recurrence 48 months postoperatively.

### DISCUSSION

Reconstruction of the trachea or bronchus has progressed remarkably in the last decade as a surgical technique for the preservation of lung function and urgent airway dilatation in patients with severe stenosis of the airway. Previously, emergency radical operation or blind intubation with a slender tracheal tube beyond the stenotic portion were necessary. In emergency surgery, tracheal intubation under local anesthesia with the patient awake was performed because of the difficulties with lung ventilation after the administration of muscle relaxant. These techniques were burdensome not only for patients but also for anesthesiologists. With blind intubation with a tracheal tube, there was the possibility of massive bleeding causing fatal complications and safer management of severe stenosis of airway was required.

Since 1981, we have treated patients with benign or malignant lesions with endoscopic Nd:YAG laser treatment. The indications included palliative irradiation for airway reopening and hemostasis in unresectable advanced disease, airway reopening before and after surgical treatment and curative irradiation for superficial carcinomas. The effects of palliation of the airway have previously been reported [5–7]; however, few reports have documented the significance of endoscopic Nd:YAG laser treatment in the perioperative period of tracheobronchoplastic surgery.

In this article, we found significant results with preoperative irradiation in patients with tracheal stenosis. Endoscopic Nd:YAG laser treatment could reopen the tracheal lumen preoperatively and contribute to the improvement of the general condition of the patients. Further, this procedure permitted the surgeon to confirm the distal margin of tumor and to safely intubate under routine general anesthesia with the use of muscle relaxant. Endoscopic Nd:YAG laser treatment is an effective preoperative procedure in patients with severe tracheal stenosis. However, the long-term survival demonstrated in this study is considered to be due to the effect of surgery and the histologic type of cancer instead of the effect of endoscopic treatment.

Endoscopic Nd:YAG laser treatment is also effective in the preoperative period in patients with hilar type bronchogenic carcinomas, because it allowed better assessment of the possible resection margins before sleeve lobectomy was performed. Stenoses due to granulation tissue formation of bronchial end-to-end anastomoses can be reopened by Nd:YAG laser treatment in patients undergoing sleeve lobectomy, and its effect is maintained for a long period when the granulomatous tissue is confined within 10 mm in longitudinal width. Endoscopic Nd:YAG laser treatment is recommended before thoracotomy is performed to remove stenosis of the bronchial anastomosis. Kato and co-workers [8] reported the significance of preoperative laser photodynamic therapy in combination with surgery for lung cancer, and this article stressed the importance of Nd:YAG laser therapy in the management of tracheoplasty.

As previously reported [9,10], endoscopic Nd:YAG laser treatment can be associated with major complications including massive bleeding or perforation if not performed properly. Nd:YAG laser treatment must be confined to a lesion within cartilaginous rings. In a sleeve lobectomy for patients with granulomatous stenosis at the end-to-end anastomosis of the bronchus, endoscopic Nd:YAG laser treatment should be performed with consideration of mediastinal anatomy and the site of both the anastomosis and pulmonary artery. A tracheobronchial stenting procedure has been reported as an additional endoscopic treatment alternative before or after Nd:YAG laser [11]. We have not yet applied stenting in this study; however, stenting is considered to be useful for stenosis of bronchial anastomosis in sleeve lobectomy.

The optimal interval between endoscopic Nd:YAG laser treatment and tracheobronchoplasty remains uncertain; however, we demonstrated that bronchial epithelium damaged with 150 J of irradiation could heal within 2 weeks. It may be considered that tracheobronchoplasty can be performed 2 weeks after the last session of Nd:YAG laser treatment. Preoperative management, including airway cleaning by nebulizer and systemic administration of antibiotics, is required.

In conclusion, endoscopic Nd:YAG laser treatment is considered to be very effective for emergency airway reopening, confirmation of the distal margin of tumor, and safe tracheal intubation as the perioperative management of tracheoplasty in patients with stenoses of trachea or mainstem bronchus.

### *Acknowledgments*

This work was supported for cancer research by the Ministry of Health and Welfare of Japan.

### *References*

[1] Pearson, F. G., Todd, T. R. J., Cooper, J. D. (1984). Experience with primary neoplasms of the trachea and carina, *J Thorac Cardiovasc Surg,* **88**, 511–516.

[2] Tsuchiya, R., Goya, T., Naruke, T., *et al.* (1990). Resection of tracheal carina for lung cancer. Procedure, complications and mortality, *J Thorac Cardiovasc Surg,* **99**, 779–787.

[3] Yamaguchi, Y. (1985). Progress of tracheobronchoplasty—present states and their problems, *J Jpn Surg Soc,* **86**, 651–656 [in Japanese].

[4] Fujisawa, T., Yamaguchi, Y., Baba, M., *et al.* (1990). Endoscopic Nd:YAG laser surgery on malignant and benign lesions of the trachea and carina, *Jpn J Surg,* **20**, 650–659.

[5] Dumon, J. F., Reboud, E., Garbe, L., *et al.* (1982). Treatment of tracheobronchial lesions by laser photoresection, *Chest,* **81**, 278–284.

[6] Arita, M., Yamaguchi, Y., Fujisawa, T. (1987). A clinical investigation on the indication of Nd-YAG laser irradiation for malignant diseases causing central airway stenosis, *J Jpn Assoc Thorac Surg,* **35**, 283–289 [in Japanese].

[7] Kvale, P. A., Eichenhorn, M. S., Radke, J. R., *et al.* (1985). YAG laser photoresection of lesions obstructing the central airways, *Chest,* **87**, 283–288.

[8] Kato, H., Konaka, C., Ono, J., *et al.* (1985). Preoperative laser photodynamic therapy in combination with operation in lung cancer, *J Thorac Cardiovasc Surg,* **90**, 420–429.

[9] Fujisawa, T., Baba, M., Saitoh, Y., *et al.* (1988). Side effects of endoscopic Nd:YAG laser treatment in malignant diseases invading central airway, *J Jpn Soc Bronchol,* **10**, 3–9 [in Japanese].

[10] Dierkesmann, R., Huzly, A. (1987). Side effects of endobronchial laser treatment, *Endoscopy,* **17**, 49–53.

[11] Dumon, J. F. (1990). A dedicated tracheobronchial stent, *Chest,* **97**, 328–332.

